# Identification of transcription factors and construction of a novel miRNA regulatory network in primary osteoarthritis by integrated analysis

**DOI:** 10.1186/s12891-021-04894-2

**Published:** 2021-12-02

**Authors:** Ying Jiang, Yi Shen, Liyan Ding, Shengli Xia, Liying Jiang

**Affiliations:** 1 Department of Epidemiology, School of Public Health, Nantong University, Nantong, Jiangsu Province P. R. China; 2 Department of Orthopedics, Shanghai University of Medicine & Health Sciences Affiliated Zhoupu Hospital, Shanghai, P. R. China; 3 Jiading District Central Hospital, Shanghai University of Medicine & Health Sciences, Shanghai, P. R. China

**Keywords:** Osteoarthritis, Circulating microRNAs, RNA sequencing, Bioinformatics analysis

## Abstract

**Backgrounds:**

As osteoarthritis (OA) disease-modifying therapies are not available, novel therapeutic targets need to be discovered and prioritized. Here, we aim to identify miRNA signatures in patients to fully elucidate regulatory mechanism of OA pathogenesis and advance in basic understanding of the genetic etiology of OA.

**Methods:**

Six participants (3 OA and 3 controls) were recruited and serum samples were assayed through RNA sequencing (RNA-seq). And, RNA-seq dataset was analysed to identify genes, pathways and regulatory networks dysregulated in OA. The overlapped differentially expressed microRNAs (DEMs) were further screened in combination with the microarray dataset GSE143514. The expression levels of candidate miRNAs were further validated by quantitative real-time PCR (qRT-PCR) based on the GEO dataset (GSE114007).

**Results:**

Serum samples were sequenced interrogating 382 miRNAs. After screening of independent samples and GEO database, the two comparison datasets shared 19 overlapped candidate micRNAs. Of these, 9 up-regulated DEMs and 10 down-regulated DEMs were detected, respectively. There were 236 target genes for up-regulated DEMs and 400 target genes for those down-regulated DEMs. For up-regulated DEMs, the top 10 hub genes were *KRAS*, *NRAS*, *CDC42*, *GDNF*, *SOS1*, *PIK3R3*, *GSK3B*, *IRS2*, *GNG12*, *and PRKCA*; for down-regulated DEMs, the top 10 hub genes were *NR3C1*, *PPARGC1A*, *SUMO1*, *MEF2C*, *FOXO3*, *PPP1CB*, *MAP2K1*, *RARA*, *RHOC*, *CDC23*, *and CREB3L2*. Mir-584-5p-*KRAS*, mir-183-5p-*NRAS*, mir-4435-*PIK3R3*, and mir-4435-*SOS1* were identified as four potential regulatory pathways by integrated analysis.

**Conclusions:**

We have integrated differential expression data to reveal putative genes and detected four potential miRNA-target gene pathways through bioinformatics analysis that represent new mediators of abnormal gene expression and promising therapeutic targets in OA.

**Supplementary Information:**

The online version contains supplementary material available at 10.1186/s12891-021-04894-2.

## Background

Osteoarthritis (OA), the most common form of arthritis, is a degenerative joint disease characterized by progressive articular cartilage and a leading cause of disability in the elderly [1, 2]. OA affects more than 300 million people according to the recent Global Burden of Disease study 2017, which is the third most rapidly rising condition associated with disability after diabetes and dementia [1, 3]. It is estimated that nearly 400 million people will suffer from OA by 2030 in China [4]. The etiology of OA is affected by factors including age, female, obesity, occupational exposure to high levels of joint loading activity, smoking status and family history, and the molecular pathophysiology is still poorly understood [4]. Epidemiological studies have reported the heritability of >40% at individual skeletal sites, while genome-wide association studies (GWAS) have revealed that OA is a heterogeneous chronic disease with multiple genetics [5]. As such, the disease is genetically complex and multi-factorial.

MicroRNA (miRNA) are a group of universally present and multifunctional small non-coding RNAs with 22–28 bases encoded by endogenous genes [6, 7], and participate in many biological processes such as cell proliferation, differentiation, apoptosis [8]. Recent breakthroughs have revealed numerous examples of miRNAs involvement in normal development and disease [9]. As one of the epigenetic mechanisms, miRNAs play a significant role in OA pathogenesis, such as cartilage homeostasis, extracellular matrix regulation, bone metabolism, apoptosis and inflammation [10]. And, much attention has recently focused on miRNA because increasing evidence indicates that miRNA affect gene transcription through a number of regulatory processes [10].

Mature miRNAs are structurally stable and essential regulators of many physiological processes. Several studies have identified numerous differentially expressed microRNAs (DEMs) between OA joint tissues and controls [11, 12]. Presumable studies demonstrated the role of miRNAs as potential biomarkers for the assessment of diagnosis and prognosis in OA [13, 14]. Nakamura et al. showed that miR-181a-5p induces articular cartilage degeneration by promoting inflammation, cartilage catabolism and apoptosis/cell death [15]. Ntoumou et al. identified that a three-miRNA signature, hsa-miR-140-3p, hsa-miR-681-3p and hsa-miR-33b-3p in OA patients, which were involved in the regulation of OA metabolism [16]. Presently, medical care is mainly based on alleviating pain symptoms and there is still a lack of effective means for the treatment of disease. It is necessary to fully understand the molecular and cellular pathways of OA and novel breakthrough in the sector of early diagnosis and treatment of OA.

In the present study, we performed miRNA profiling using high-density miRNA-arrays in peripheral blood leukocytes (PBL) and compared the expression profiles of miRNAs between OA patients and controls. Then, overlapped DEMs were screened by downloading the miRNAs expression data from the Gene Expression Omnibus (GEO) database (GSE143514). We created a molecular profile of the OA transcriptome and performed functionl enrichment analyses to elucidate perturbed molecular functions and pathways in OA, including transcription factors (TFs)-DEMs, DEMs-target genes, TFs of target genes, and DEMs-hub genes network. Hub genes were further performed to validate the expression levels based on the GSE114007 dataset. Finally, we developed a high-resolution molecular profile of OA and elucidated a novel miRNA regulatory network of OA pathogenesis.

## Materials and methods

### Sample collection

A two-stage study was conducted in this study. In the first RNA-sequencing screening stage, blood samples were collected form 3 OA patients and 3 healthy controls (age over 40 years old) in March 2018.

The inclusion criteria for OA were presented as follows: (i) Complete basic personal information; (ii) Conforming to the diagnosis criteria of disease [17]. The normal controls were recruited from the survey that had no signs or symptoms of arthritis or joint diseases. Secondary OA patients such as inflammatory arthritis, rheumatoid, bone fracture and developmental dysplasia were excluded. MicroRNA expression dataset was downloaded from the GEO database as another screening stage under the accession number GSE143514.

Subsequently, in the second stage validation, the expression levels of hub genes were further verified using quantitative reverse transcription polymerase chain reaction (qRT-PCR) based on the GSE114007 dataset.

### Library preparation and sequencing

White blood cells are extracted by centrifugation at 1500 g for 20 min with 5 ml whole blood. Following the manufacture’s protocol, we used TRIzol (Invitrogen, Carlsbad, CA, USA) to isolate total RNA.

RNA-seq was operated by Gminix Biotechnology Co., Ltd. (Shanghai, China) using 150 ng of total RNA as input, and the results was evaluated by an Illumina HiSeq 2500 sequencing platform with 10 M reads (Illumina, San Diego, CA, USA). Hisat2 [18] were used to compare DEMs, and feature Counts [19] were adopted to annotate and quantify miRNAs. MiRNAs expression was normalized by principal component analysis (PCA) using R software. Then, using the DESeq2 package in R (http://bioconductor.org/packages/release/bioc/html/DESeq2.html), counts were analyzed and DEMs were screened. Herein, miRNAs with an absolute value fold changes (FC) ≥ 1.2 and *P* < 0.05 were considered as the significance.

### Data Collection for GSE143514 and Identification of candidate DEMs

The GEO database (https://www.ncbi.nlm.nih.gov/gds) is an international public repository that provides high-throughput gene expression data [20]. The GEO dataset GSE143514, including five OA patients and three controls were screened [21]. All samples were detected by the platform HiSeq x-ten platform (Illumina). For data processing, the raw data were downloaded (https://www.ncbi.nlm.nih.gov/geo/query/acc.cgi?acc=GSE143514). The limma software package in R (https://bioconductor.org/packages/release/bioc/html/limma.html) was employed to screen DEMs for OA patients and healthy controls. The absolute value fold changes (FC) ≥1.2 and *P* < 0.05 were set as the threshold for identifying DEMs.

Respectively, the volcano maps of DEMs in the internal dataset and GSE143514 database were drawn using GraphPad Prism. Venn diagram was utilized to analyze the overlapped DEMs between the internal dataset and GEO dataset (GSE143514), and these miRNAs were considered as candidate DEMs.

### Prediction of Upstream Transcription Factors and Downstream Target Genes of DEMs

FunRich, an independent software tool for the analysis of gene and protein functional enrichment and interaction networks [22], was employed to predict the potential upstream TFs of candidate DEMs. Finally, we presented the top 10 transcription factors. The *P* value<0.05 was considered statistically significant.

Based on the above primary analysis of candidate DEMs, target genes were synchronously predicted using miRTarBase 7.2 (http://www.targetscan.org/vert_72/), miRDB (http://mirdb.org/), miRwalk (http://mirwalk.umm.uni-heidelberg.de/) and MicroT-CDS (http://diana.imis.athena-innovation.gr/DianaTools/index.php?r=microT_CDS/index). For a relatively robust selection of target genes, the overlapped target genes from the above four databases were detected by Venn diagram analysis.

### Functional Analysis of Target Genes

Bioinformatics analysis was used to investigate the involved pathways and target genes for the above miRNAs. Gene Ontology (GO) is a comprehensive gene annotation tool that provides information on gene function from molecular level to organism point, including biological process (BP), molecular function (MF), and cellular component (CC) [23]. Kyoto Encyclopedia of Genes and Genomes (KEGG) is a comprehensive database that broadly interprets biological pathways and identifies the functions of candidate DEMs in biological pathways [24]. In our study, the database for annotation, visualization, and integrated discovery (DAVID) v6.8 (https://david.ncifcrf.gov) online tool was performed for candidate DEMs enrichment analysis. *P* < 0.05 for GO analysis and *P* < 0.1 and count>2 for the KEGG pathway were considered for further analysis.

### Construction of PPI Network and Screening of Hub Genes

Investigation on the key miRNA and target genes protein-protein interaction (PPI) network is beneficial for the exploration of molecular mechanism of the disease. The Search Tool for the Retrieval of Interacting Genes/Proteins (STRING) online database (http://stringdb.org/) was employed to analyze the interaction between miRNA and target genes. PPI node pairs with a combined score ≥ 0.4 were selected for further analysis. We further matched up-regulated target genes with down-regulated target genes and down-regulated miRNA with up-regulated genes. Cytoscape 3.7.1 was used to visualize the network and annotate genes within enriched pathways in the network. The hub genes were screened by CytoHubba, a plugin of Cyoscape, according to the degree. The top 30 target genes were selected using the Maximal Clique Centrality (MCC).

### Expression Analysis of Hub Genes based on GSE114007

For further verification analysis, the GSE114007 database was introduced to analyze the expression level of hub genes. The dataset was performed on the platform GPL11154 Illumina HiSeq 2000 and GPL18573 Illumina NextSeq 500, including 20 OA knee cartilages and 18 normal cartilage tissues. DEMs between OA and control group were analyzed by Student’s *t*-test. The hub genes must conform to the following criteria: (i) the relationship of gene regulation is definitely consistent with previous studies; (ii) the *P* value should be less than 0.05.

### Statistical analysis

Body mass index (BMI) was calculated by dividing the weight (kg) by the squared height (m^2^), and the participants were divided into four categories according to the Working Group on Obesity in China recommended criteria (Underweight: BMI < 18.5 kg/m2; Normal: 18.5 ≤ BMI < 24; Overweight: 24 ≤ BMI < 28; and General obesity: BMI ≥ 28), [25]. A value of *P* < 0.05 was considered statistically significant. All analysis was performed with SPSS 22.0 (IBM Corp) and GraphPad Prism version 9.0 (GraphPad Software, lnc).

## Results

**Fig. 1:**
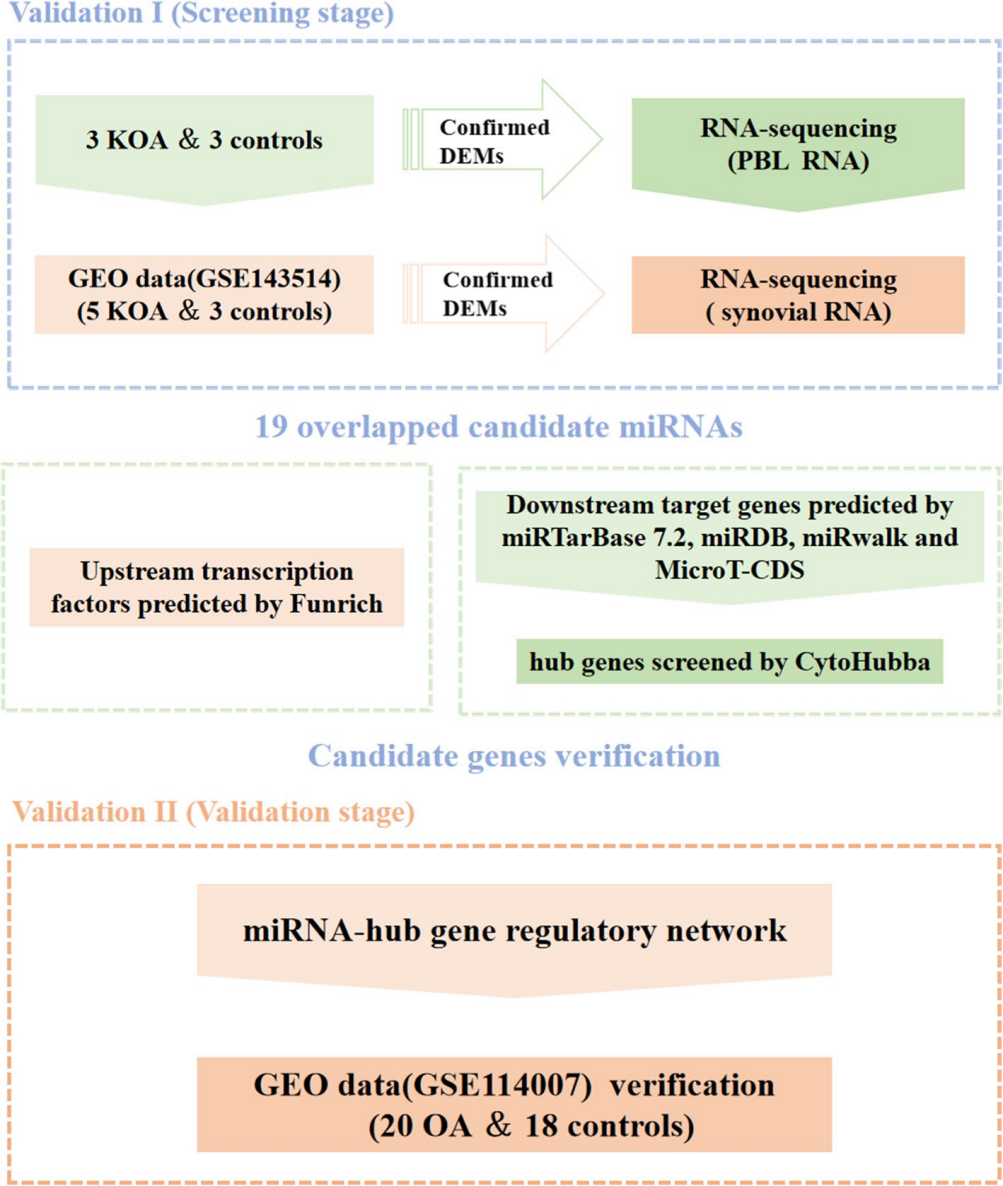
Flow chart of constructing the miRNA regulatory network in OA

A schematic diagram of the study design is displayed in Fig. 1, and the characteristics of the internal database are shown in Table 1.

**Table 1:** Characteristics of the subjects enrolled for miRNA expression analysis in the study

Variables	Case (n = 3)	Control (n = 3)	***P***
Age (mean ± SD)^a^	64.72 ± 7.81	61.73 ± 7.59	0.600
Gender, N(100%)			
Male	2 (66.67)	2 (66.67)	–
Female	1 (33.33)	1 (33.33)	
BMI			
< 24	0	0	–
24 ≤ BMI < 28	2 (66.67)	2 (66.67)	
≥ 28	1 (33.33)	1 (33.33)	
Smoking status			
Current	0	0	0.532
Ever	1 (33.33)	2 (66.67)	
Never	2 (66.67)	1 (33.33)	
Drinking status			
Current	0	0	0.532
Ever	1 (33.33)	2 (66.67)	
Never	2 (66.67)	1 (33.33)	

^a^Median age in all subjects

*BMI* body mass index

### Identification of DEMs

**Fig. 2:**
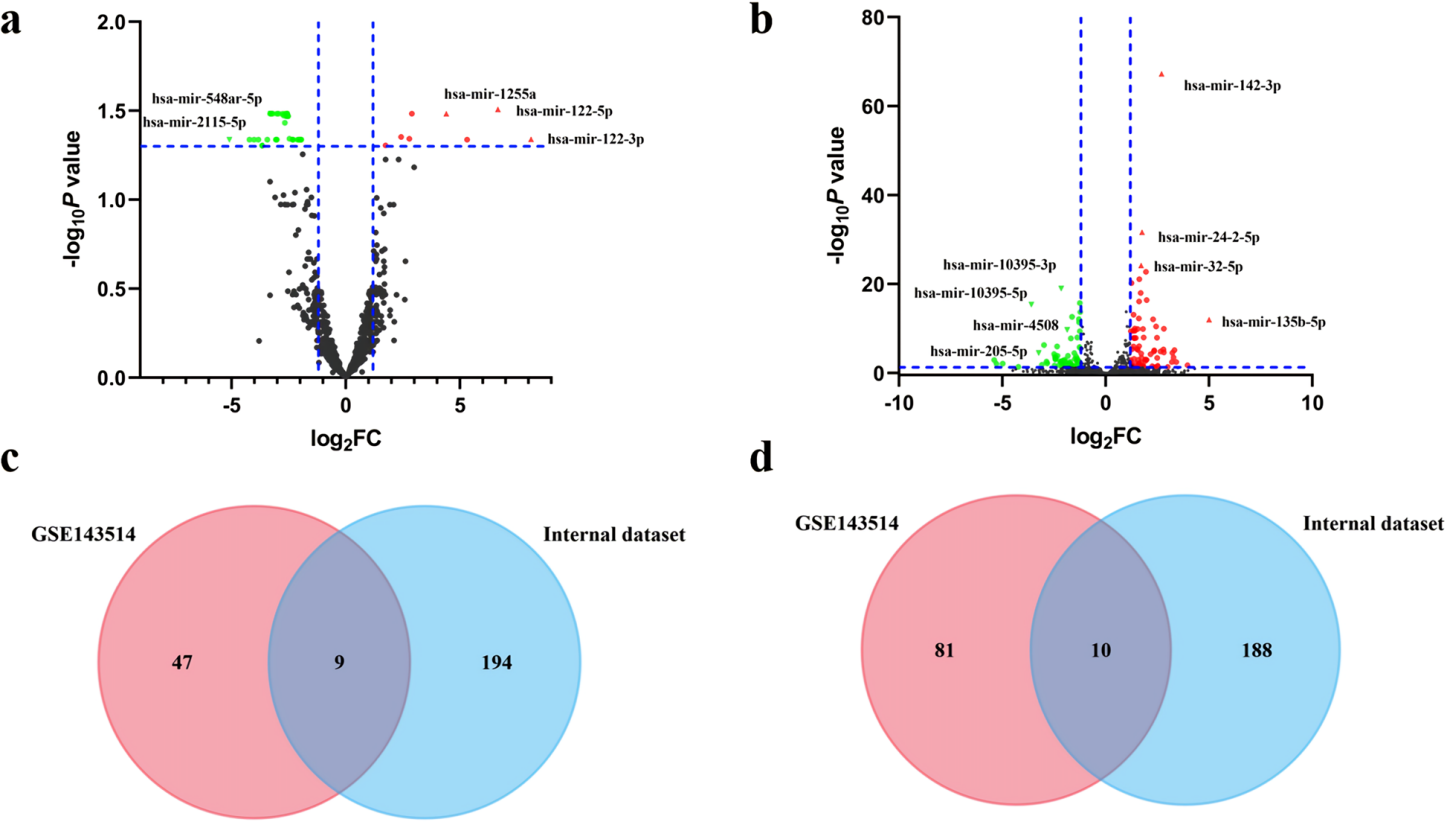
Identification of differentially expressed miRNAs (DEMs). **a** DEMs of the GSE143514 database. **b** DEMs of the internal database. **c** intersection of the two databases with down-regulated DEMs. **d** intersection of the two databases with up-regulated DEMs

Six serum samples were sequenced based on the selection criteria of adjusted *P* < 0.05 and |logFC| > 1.2. Briefly, a total of 382 DEMs were identified from the internal database, including 194 up-regulated miRNAs and 188 down-regulated miRNAs. Similarly, 128 DEMs (47 up-regulated and 81 down-regulated) were identified in the GSE143514 dataset. The volcano maps of the two databases were presented in Fig. 2a and b, respectively.

` After screening of independent samples and GEO database, the two comparison datasets shared 19 overlapped candidate miRNAs. The co-existing DEMs were displayed in Fig. 2c and d via Venn diagrams. Among these DEMs, we detected 9 up-regulated DEMs (hsa-mir-34a-5p, hsa-mir-4777-3p, hsa-mir-129-2-3p, hsa-mir-152-5p, hsa-mir-200a-3p, hsa-mir-6503-5p, hsa-mir-138-5p, hsa-mir-501-5p, and hsa-mir-342-5p) and 10 down-regulated DEMs (hsa-mir-185-5p, hsa-mir-4732-3p, hsa-mir-4732-5p, hsa-mir-3143, hsa-mir-584-5p, hsa-mir-4435, hsa-mir-1246, hsa-mir-151a-3p, hsa-mir-183-5p, and hsa-mir-501-3p).

### Prediction of Transcription Factors of DEMs

**Fig. 3:**
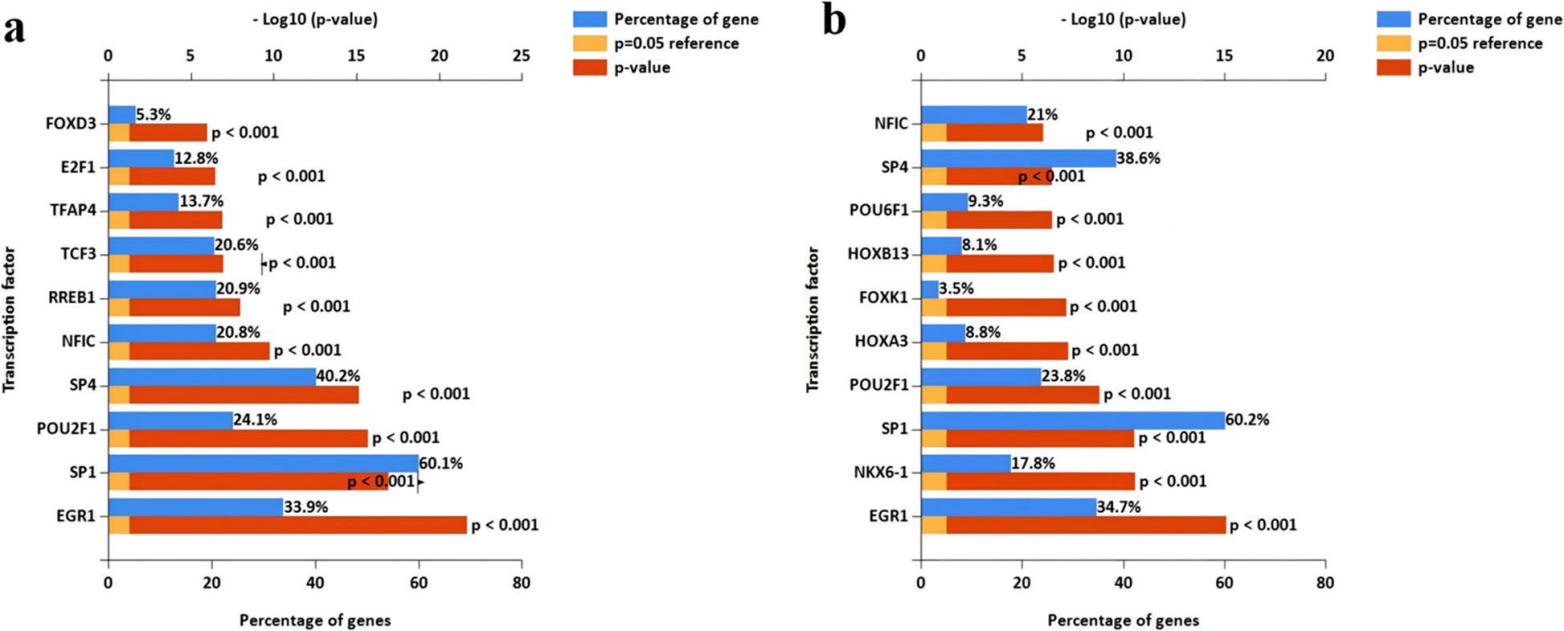
Predicted transcription factors of DEMs. **a** top ten upstream transcription factors of up-regulated DEMs. **b** top ten upstream transcription factors of down-regulated DEMs

In the study, we used FunRich software to predict the upstream transcription factors of up-regulated and down-regulated DEMs and presented the top 10 transcription factors in Fig. 3a and b, respectively. For up-regulated DEMs, the top 10 transcription factors include EGR1, SP1, POU2F1, SP4, NFIC, RREB1, TCF3, TFAP4, E2F1, and FOXD3. For down-regulated DEMs, the top 10 transcription factors were EGR1, NKX6–1, SP1, POU2F1, HOXA3, FOXK1, HOXB13, POU6F1, SP4, and NFIC.

### Prediction of Target Genes of DEMs

**Fig. 4:**
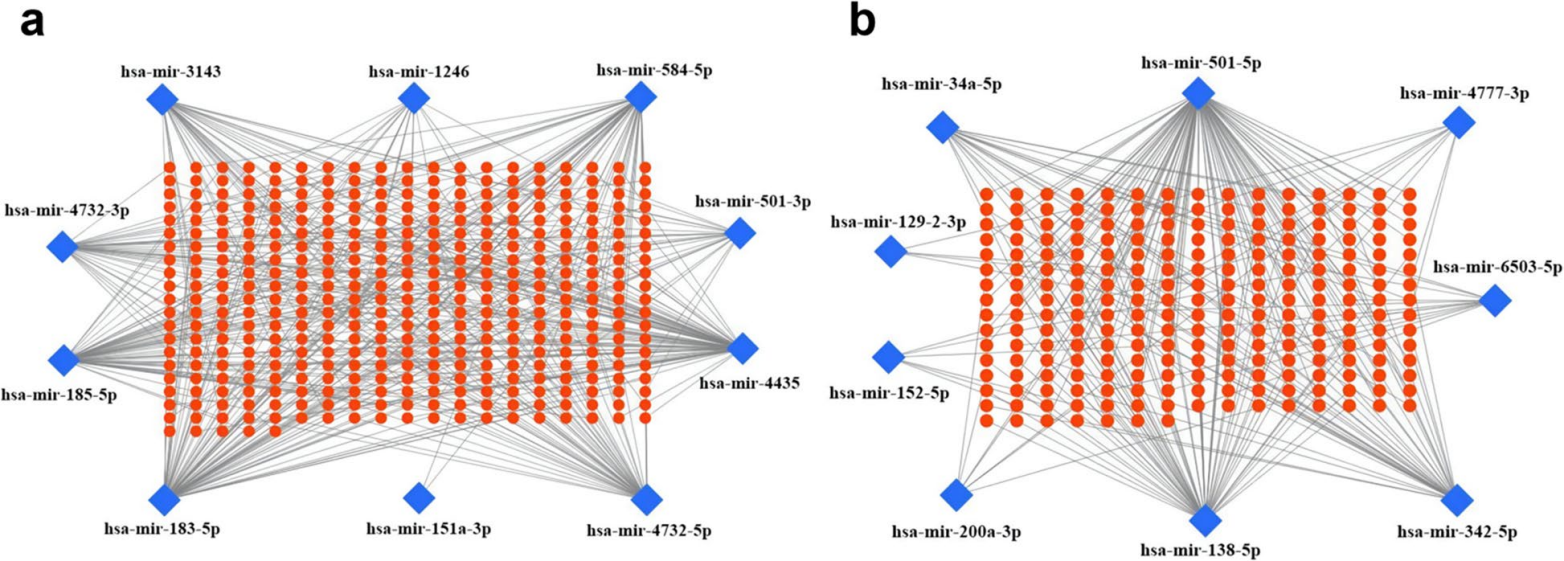
Potential target genes of DEMs predicted by databases. **a** up-regulated miRNA-target gene network. **b** down-regulated miRNA-target gene network

To identify the miRNA-mRNA regulatory axes involved in OA, we used miRTarBase 7.2, miRDB, miRwalk and MicroT-CDS databases to predict the target genes of DEMs. As shown in Table 2, we explored the target genes of miRNAs based on different databases. The overlapped target genes from the above four databases were screened by Venn diagram analysis and presented in Supplementary Table 1. There were 236 target genes for up-regulated DEMs and 400 ones for down-regulated DEMs. The DEMs-target gene network were plotted to more intuitively explore the relationship between DEMs and target genes (Fig. 4a and b).

**Table 2:** miRNA-target genes count

Attribute	miRNA	Target genes			
		miRDB	miRwalk	targetscan	microT-CDS
Down-regulated DEMs	hsa-mir-151a-3p	220	2930	112	130
	hsa-mir-183-5p	565	3863	509	832
	hsa-mir-1246	407	1396	3032	861
	hsa-mir-185-5p	1137	7186	385	1406
	hsa-mir-3143	1426	579	5274	1555
	hsa-mir-4435	588	6252	5518	946
	hsa-mir-4732-3p	523	5718	4112	338
	hsa-mir-4732-5p	186	8995	2607	510
	hsa-mir-501-3p	289	4812	200	733
	hsa-mir-584-5p	402	6119	2668	704
Up-regulated DEMs	hsa-mir-129-2-3p	433	6230	56	491
	hsa-mir-138-5p	657	5556	704	893
	hsa-mir-152-5p	459	2688	3817	147
	hsa-mir-200a-3p	1088	1466	109	1509
	hsa-mir-342-5p	385	6345	3350	411
	hsa-mir-34a-5p	899	6100	186	1108
	hsa-mir-4777-3p	297	1754	2564	307
	hsa-mir-501-5p	615	7996	3892	902
	hsa-mir-6503-5p	287	4038	1894	400

*DEMs* differentially expressed microRNAs

### Enrichment Analysis of Target Genes

**Fig. 5:**
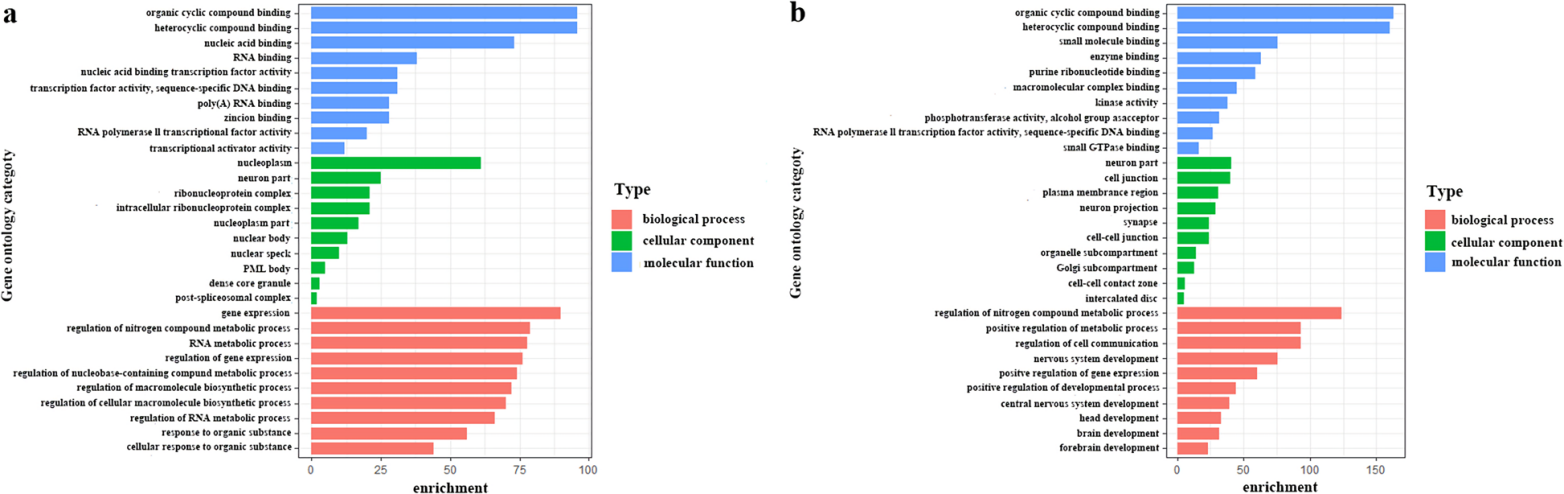
GO annotation analysis for the target genes of DEMs in the biological process, cellular component, and molecular function. **a** GO analysis for up-regulated DEG. **b** GO analysis for down-regulated DEGs

We further explored biological functions of those candidate target genes through the DAVID database. The top 10 GO enrichment results of up-regulated and down-regulated target genes were shown in Fig. 5a and b, respectively. In the BP group, the up-regulated genes were significantly enriched in gene expression, regulation of nitrogen compound metabolic process, RNA metabolic process, regulation of gene expression, and regulation of nucleobase-containing compound metabolic process, while the down-regulated genes were enriched in regulation of nitrogen compound metabolic process, positive regulation of metabolic process, regulation of cell communication, and nervous system development, etc. In the CC group, the up-regulated genes were significantly enriched in nucleoplasm, neuron part, ribonucleoprotein complex, intracellular ribonucleoprotein complex, and nucleoplasm part, while the down-regulated genes were mainly involved the neuron part, cell junction, plasma membrane region, neuron projection, and synapse, etc. In the MF group, the up-regulated genes were significantly enriched in organic cyclic compound binding, heterocyclic compound binding, nucleic acid binding, RNA binding, and nucleic acid binding transcription factor activity, while the down-regulated genes were mainly involved in organic cyclic compound binding, heterocyclic compound binding, small molecule binding, enzyme binding, and purine ribonucleotide binding, etc.

**Fig. 6:**
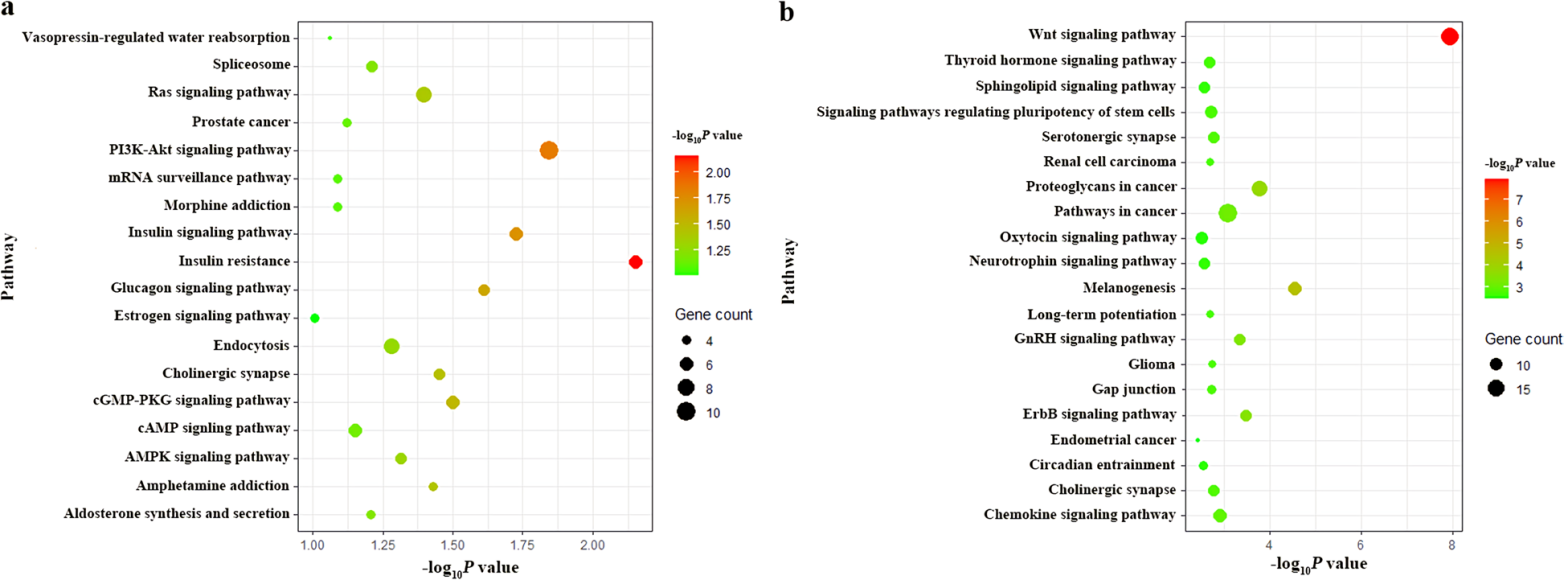
KEGG pathway analyses for the target genes of DEMs. **a** KEGG pathway analysis for up-regulated DEMs. **b** KEGG pathway analysis for down-regulated DEMs

Subsequently, we conducted a KEGG pathway enrichment analysis on these target genes of DEMs. As shown in Fig. 6a and b, we identified 18 critical pathways for up-regulated target genes and the top 20 pathways for down-regulated target genes, respectively. KEGG analysis suggested that the up-regulated genes were significantly enriched in insulin resistance, PI3K-Akt signaling pathway, and insulin signaling pathway, while the down-regulated genes mainly participated in the Wnt signaling, cancer, melanogenesis, and proteoglycans in cancer, etc.

### Construction of DEM-Hub Genes Network

**Fig. 7:**
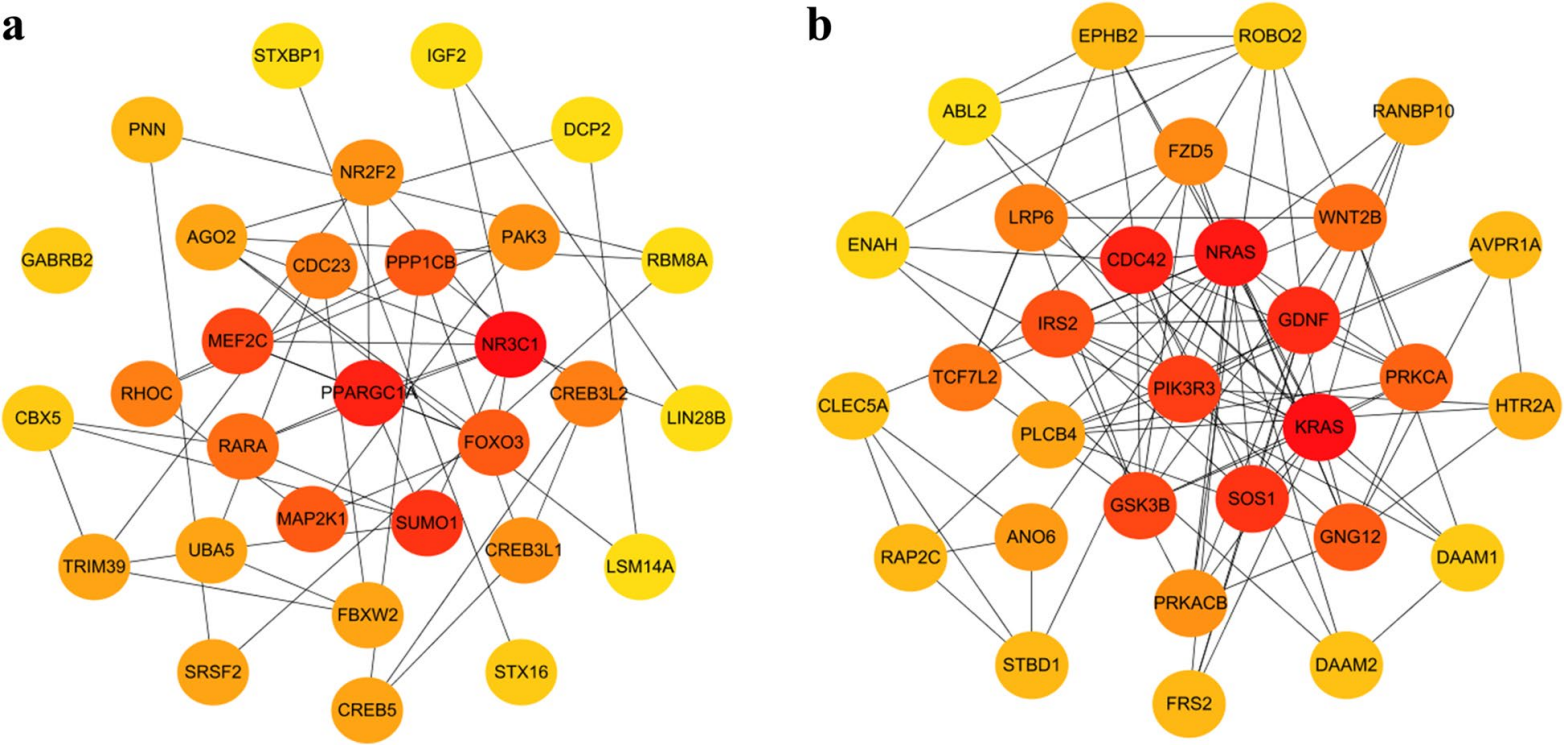
Identification of the hub genes for DEMs in the PPI network. **a** PPI network of the top 30 hub genes for up-regulated DEMs. **b** PPI network of the top 30 hub genes for down-regulated DEMs

**Fig. 8:**
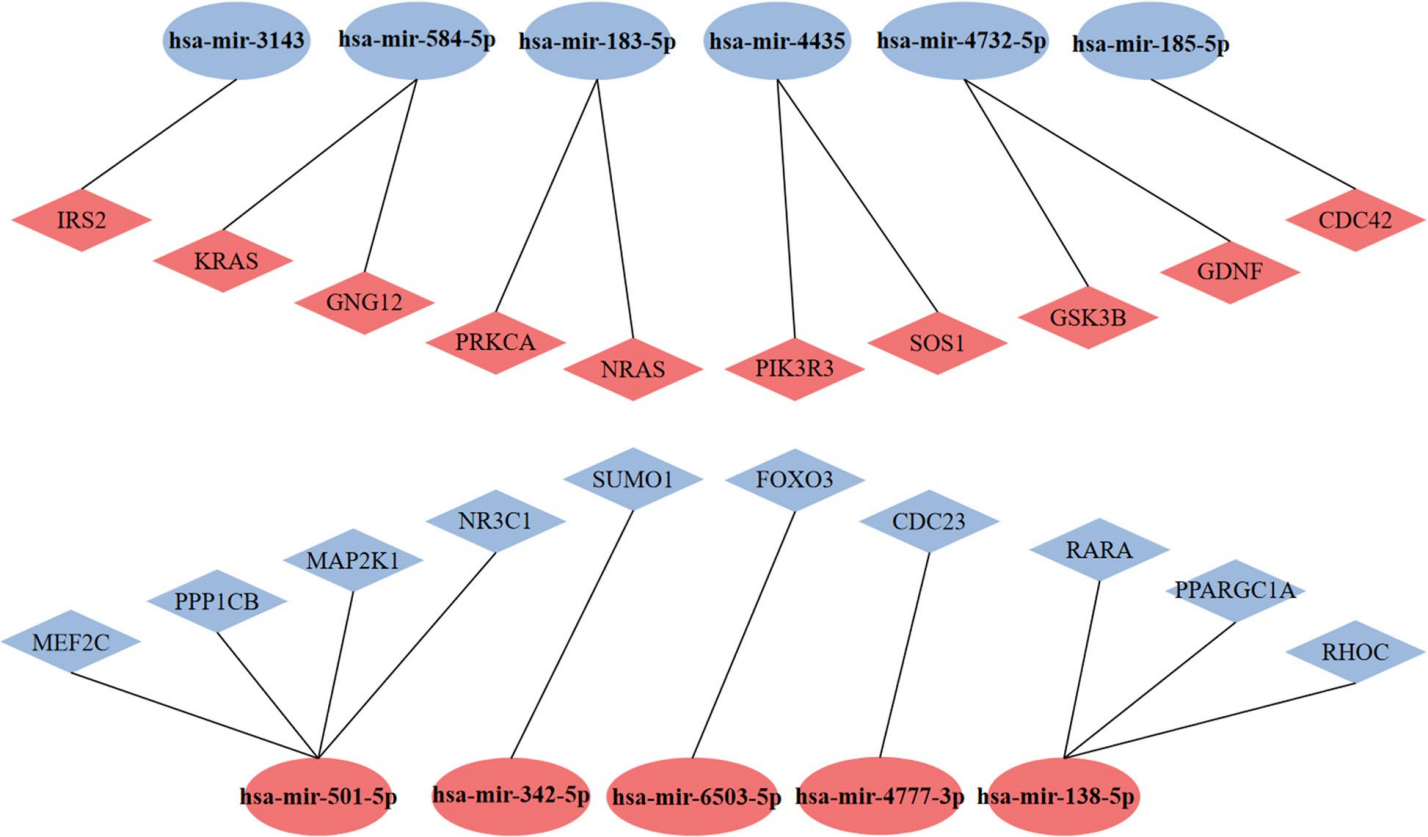
Identification of the hub genes for DEMs in the PPI network. Diamond represents genes, ellipse represents miRNAs. Red represents up-regulated DEMs and hub genes, Blue represents down-regulated DEMs and hub genes

The top 30 hub genes for up-regulated and down-regulated DEMs are shown in Fig. 7a and b, respectively. For up-regulated DEMs, the top 10 hub genes were KRAS, NRAS, CDC42, GDNF, SOS1, PIK3R3, GSK3B, IRS2, GNG12 and PRKCA. For down-regulated DEMs, the top 10 hub genes were NR3C1, PPARGC1A, SUMO1, MEF2C, FOXO3, PPP1CB, MAP2K1, RARA, RHOC, CDC23, and CREB3L2(Table 3). We constructed the candidate miRNA-hub genes regulatory network to precisely explore the molecular mechanism of these DEMs, which was presented in the form of a diagram (Fig. 8).

**Table 3:** Top 10 hub genes of the significantly up-regulated and down-regulated DEMs in the PPI network ranked by MCC

ID	Down-regulated DEMs		Up-regulated DEMs	
	Gene Symbol	Score	Gene Symbol	Score
1	KRAS	253	NR3C1	23
2	NRAS	237	PPARGC1A	22
3	CDC42	132	SUMO1	17
4	GDNF	119	MEF2C	15
5	SOS1	92	FOXO3	12
6	PIK3R3	79	PPP1CB	12
7	GSK3B	72	MAP2K1	12
8	IRS2	58	RARA	10
9	GNG12	57	RHOC	9
10	PRKCA	42	CDC23	9
11			CREB3L2	9

*DEMs* differentially expressed microRNAs

### Validation of Hub Genes Expression

**Fig. 9:**
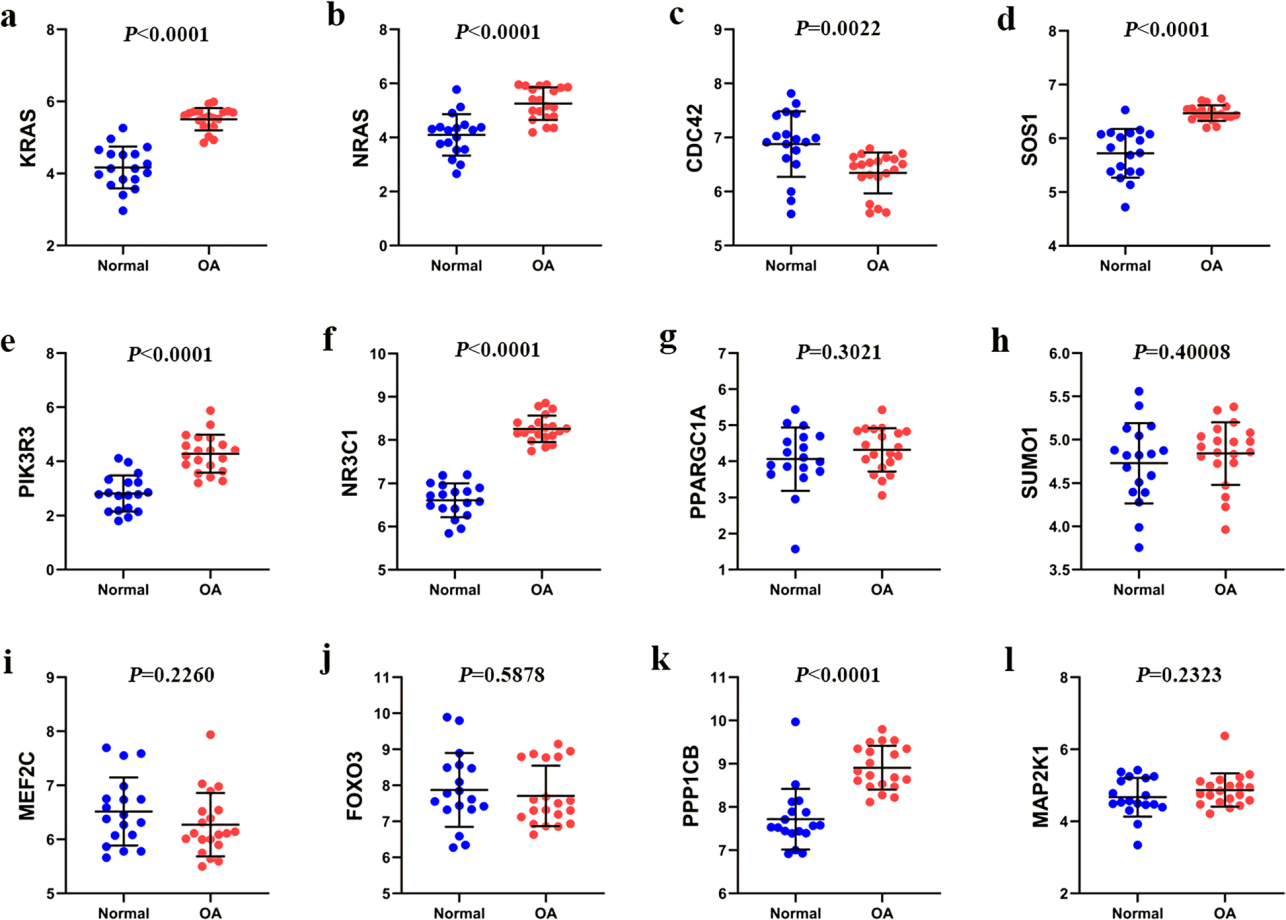
The mRNA expression of the top 12 hub genes was determined from the GSE114007 dataset (a). KRAS (b). NRAS (c). CDC42 (d). SOS1 (e). PIK3R3 f). NR3C1 (g). PPARGC1A (h). SUMO1 (i). MEF2C (j). FOXO3 (k). PPP1CB (l). MAP2K1. *P* < 0.05 was considered statistically significant

In this study, 13 hub genes with high interactions such as *KRAS*, *NRAS*, *CDC42*, *GDNF*, *SOS1*, *PIK3R3*, *NR3C1*, *PPARGC1A*, *SUMO1*, *MEF2C*, *FOXO3*, *PPP1CB* and *MAP2K1* were verified through GEO dataset GSE114007. Regrettably, GDNF is barely expressed in this database. The expression of *KRAS*, *NRAS*, *PIK3R3*, and *SOS1* continue to increase for down-regulated DEMs, which was consistent with the bioinformatics analysis (Fig. 9a-e). For up-regulated DEMs, the expression levels of *NR3C1* and *PPP1CB* were incompatible with the previous screening results; other mRNAs (*PPARGC1A*, *SUMO1*, *MEF2C*, *FOXO3 *and *MAP2K1*) were not significantly different(*P* > 0.05)(Fig. 9f-l). Thus, mir-584-5p-*KRAS*, mir-183-5p-*NRAS*, mir-4435-*PIK3R3* and mir-4435-*SOS1* were identified as four potential regulatory pathways in our study.

## Discussion

OA is the most common chronic condition associated with aging and progressive joint dysfunction and the disease with the greatest socioeconomic cost. There is a clear and urgent need to explore the aetiology and progression and support the development of disease-modifying therapies for OA. Our study provided strong evidence for the replication of previous screening integrating RNA-seq with integrated bio-informatics analysis, which may considerably increase the possibility of identifying potential biomarkers and greatly enhance the stability of our findings. To the best of our knowledge, this is the first study to comprehensively explore and identify hub miRNAs of OA from PBL of participants in Southern Chinese population. The study shed a light on plausible etiology of OA, although their biological mechanism still remains to be elucidated in the future.

In the study, we screened out 9 up-regulated DEMs and 10 down-regulated DEMs by exploring overlapped DEMs in the PBL of internal samples and synovial tissue of GSE143514 database. Among those up-regulated DEMs, mir-34a-5p could play an important role in the cell progression of osteosarcoma and could induce chondrocyte apoptosis during OA progression [26]. A previous study reported that mir-138-5p could inhibit the apoptosis and inflammation of chondrocytes and regulate cartilage phenotype and osteogenesis [27]. For down-regulated DEMs, mir-1246 could be involved in the regulation of chondrocyte cell apoptosis and IL-6 secretion from chondrocyte cells [28]. Notably, those critical miRNAs play important roles in regulating biological functions in the process of OA.

Previous studies have shown that TFs can regulate miRNA expression [29]. We found that ERG1 definitely co-exist as the top transcription factor among those up-regulated DEMs and down-regulated DEMs. EGR1 belongs to the EGR family of C2H2-type zinc-finger protein [30]. EGR1 could accelerate cartilage hyper proliferation and degeneration by regulating KLF5 expression and *β*-catenin signaling pathway [31]. Specificity protein 1(SP1), as a C2H2-type zinc-finger transcription factor, inhibits the proliferation of chondrocytes and promotes the apoptosis of chondrocytes by binding with miR-145 [32]. Eukaryotic Translation Termination Factor 1(E2F1) could regulate the potentials in modulating articular chondrocytes and participate in the regulatory mechanism of knee OA [33]. This suggest that these candidate DEMs play important roles in the pathogenesis of OA.

The expression of 4 genes (*KRAS*, *NRAS*, *PIK3R3*, *SOS1*) was consistent with that in GSE114007 dataset from the results of 12 hub genes, representing high priority candidates for therapeutic interventions. *KRAS*, a heterodimer of RAS, leads to chondrocyte growth by stimulating the MAPK/ERK1/2/Cyclin D1 signaling pathway [34]. RAS has been shown to stabilize *KRAS* levels in the cytoplasmic membrane, aggregating *KRAS* to amplify the signal output of the mitogen-activated protein kinase (MAPK) pathway [35]. As a member of the pleiotrophin, Midkine (MK) can interact with RAS and further lead to the formation of MK-RAS-KRAS complexes [36]. Then, this action stimulates the downstream MAPK/ErK1/2 signalling pathway, thereby increasing the expression of cyclin D1 and accelerating cell cycle progression [37]. Previous studies reported positive correlations between chondrocyte death by apoptosis and the severity of OA [38, 39]. Moreover, *NRAS* is an N-RAS oncogene that encodes a membrane protein travelling between the Golgi body and the plasma membrane. Actually, only a small number of studies investigated the role of NRAS in the development of OA. Huang et al. have reported that *NRAS* is an up-regulated gene in synovial tissue and associated with the MAPK pathway based on pathway analysis [40]. The MAPK pathway is the most important signal transduction system that mediates osteoarthritis cartilage injury, causing a series of reactions, such as the increased expression of matrix MMPs, chondrocyte apoptosis and cartilage destruction [40]. As a key subunit of the IAPI3K protein complex [41], *PIK3R3* regulates anabolism effector T cell differentiation, T cell senescence, and activation-induced cell death [42]. *PIK3R3* is down-regulated in human cartilage tissue with OA and also mediates the effects of miR-1236 on chondrocyte proliferation and apoptosis [43]. Additionally, *SOS1* is a guanine nucleotide exchange factor (GEF) for RAS involved in the RAS signaling pathway [44]. Major components of RAS, including ACE, AT1R, and AT2R, are expressed in synovial tissue in humans and participate in the pathogenesis of OA [45]. Kawakami et al. confirmed that chondrocytes partially expressed AT1R and AT2R and presented increased expression with the stimulation of inflammatory cytokines IL-1 [46].

This study has several merits. First, we have systematically characterized the profile of miRNAs and mRNAs expression between OA patients and healthy controls (blood samples) integrating with public data GSE143514(synovial tissues) to screen overlapped DEMs, which contain different tissues from participants. Second, we performed miRNA-associated integrated network analysis to identify potential hub genes by bioinformatics analysis. More importantly, the acquisition of blood samples is actually feasible as a means of early screening and diagnosis compared with cartilage samples and/or synovial tissues in clinical practice.

Also, there are still some limitations need to be considered. First of all, the sample size of the integrated analysis, neither the internal data nor the GEO dataset, is sufficient to explore and verify those candidate miRNAs and key genes. Considering the veracity and credibility, further studies with larger sample size are still needed to validate the findings. And, although we employed the internal samples and the public databases to comprehensively screen the critical miRNAs and key genes, the involvement of these factors and their value as therapeutic targets need to be validated in studies on their function in vitro and in vivo.

In summary, using integrated analysis, we have detected a series of potential miRNA-target gene pathways (mir-584-5p-*KRAS*, mir-183-5p-*NRAS*, mir-4435-*PIK3R3*, and mir-4435-*SOS1*) that represent new mediators of abnormal gene expression and high priority targets for therapeutic interventions. Our findings could advance in basic understanding of risk factors and molecular mechanisms of OA disease. Screening from large populations also remains to be further elucidated and validated.

## Supplementary Information

**Additional file 1.**

## Data Availability

This manuscript contains previously unpublished data. Internal dataset has been uploaded to jianguoyun software (https://www.jianguoyun.com/p/DSPmrX8Q1Iv9CRjW4ZgE). The other datasets analyzed in the present study are available in Gene Expression Omnibus (https://www.ncbi.nlm.nih.gov/geo/).

## Abbreviations

OA: Osteoarthritis
GWAS: Genome-wide association studies
miRNA: MicroRNA
DEMs: Differentially expressed microRNAs
PBL: Peripheral blood leukocytes
GEO: Gene Expression Omnibus
GO: Gene Ontology
BP: Biological process
MF: Molecular function
CC: Cellular component
KEGG: Kyoto Encyclopedia of Genes and Genomes
PPI: Protein-protein interaction
