# Supplementary material for: Identification of transcription factors and construction of a novel miRNA regulatory network in primary osteoarthritis by integrated analysis
Source: BMC Musculoskelet Disord. 2021 Dec 2;22:1008. doi: 10.1186/s12891-021-04894-2 (PMC8641180; doi:10.1186/s12891-021-04894-2)
Supplement: Supplementary file 1 — Additional file 1. [file 12891_2021_4894_MOESM1_ESM.docx]

**Supplementary Table 1** Collection of target genes of DEMs predicted by four databases

| microRNA | Target genes |
| --- | --- |
| hsa-miR-151a-3p | NIPAL2 |
| hsa-miR-151a-3p | PANK2 |
| hsa-miR-183-5p | PIGX |
| hsa-miR-183-5p | SCYL3 |
| hsa-miR-183-5p | LHFPL2 |
| hsa-miR-183-5p | HECTD2 |
| hsa-miR-183-5p | FRS2 |
| hsa-miR-183-5p | FRMD5 |
| hsa-miR-183-5p | CELF1 |
| hsa-miR-183-5p | ABI2 |
| hsa-miR-183-5p | QKI |
| hsa-miR-183-5p | KIF2A |
| hsa-miR-183-5p | ZFPM2 |
| hsa-miR-183-5p | KLHL28 |
| hsa-miR-183-5p | REPS2 |
| hsa-miR-183-5p | FOXN2 |
| hsa-miR-183-5p | PRKACB |
| hsa-miR-183-5p | PSEN2 |
| hsa-miR-183-5p | GREM2 |
| hsa-miR-183-5p | PLCB4 |
| hsa-miR-183-5p | CHD2 |
| hsa-miR-183-5p | SMPD3 |
| hsa-miR-183-5p | C16orf72 |
| hsa-miR-183-5p | GPAM |
| hsa-miR-183-5p | FLRT3 |
| hsa-miR-183-5p | BNIP3L |
| hsa-miR-183-5p | RABGAP1L |
| hsa-miR-183-5p | RAP2C |
| hsa-miR-183-5p | FNDC3B |
| hsa-miR-183-5p | HTR2A |
| hsa-miR-183-5p | BNC2 |
| hsa-miR-183-5p | AAK1 |
| hsa-miR-183-5p | RBMS1 |
| hsa-miR-183-5p | PSD3 |
| hsa-miR-183-5p | PKD2 |
| hsa-miR-183-5p | DGKH |
| hsa-miR-183-5p | PHF3 |
| hsa-miR-183-5p | BTG1 |
| hsa-miR-183-5p | YTHDF3 |
| hsa-miR-183-5p | SPATA17 |
| hsa-miR-183-5p | SOCS6 |
| hsa-miR-183-5p | TCF12 |
| hsa-miR-183-5p | ATP2C1 |
| hsa-miR-183-5p | NRAS |
| hsa-miR-183-5p | NR3C1 |
| hsa-miR-183-5p | PLEKHA3 |
| hsa-miR-183-5p | BZW1 |
| hsa-miR-183-5p | CLVS2 |
| hsa-miR-183-5p | SPRED1 |
| hsa-miR-183-5p | STYX |
| hsa-miR-183-5p | PRUNE2 |
| hsa-miR-183-5p | CSMD1 |
| hsa-miR-183-5p | RNF138 |
| hsa-miR-183-5p | SEL1L |
| hsa-miR-183-5p | KCNK2 |
| hsa-miR-183-5p | EEF2 |
| hsa-miR-183-5p | LRP6 |
| hsa-miR-183-5p | PLAG1 |
| hsa-miR-183-5p | CX3CL1 |
| hsa-miR-183-5p | CTDSP1 |
| hsa-miR-183-5p | RAB30 |
| hsa-miR-183-5p | ACVR2B |
| hsa-miR-183-5p | LMO3 |
| hsa-miR-183-5p | ROBO2 |
| hsa-miR-183-5p | ARHGAP6 |
| hsa-miR-183-5p | TCF7L2 |
| hsa-miR-183-5p | VANGL1 |
| hsa-miR-183-5p | L3MBTL3 |
| hsa-miR-183-5p | ZNF770 |
| hsa-miR-183-5p | DAAM1 |
| hsa-miR-183-5p | NPAS3 |
| hsa-miR-183-5p | PRKCA |
| hsa-miR-185-5p | TEAD1 |
| hsa-miR-185-5p | SORL1 |
| hsa-miR-185-5p | SLC8A1 |
| hsa-miR-185-5p | LCOR |
| hsa-miR-185-5p | CDC42EP3 |
| hsa-miR-185-5p | HP1BP3 |
| hsa-miR-185-5p | TANC2 |
| hsa-miR-185-5p | SLC16A2 |
| hsa-miR-185-5p | CNTD1 |
| hsa-miR-185-5p | THRA |
| hsa-miR-185-5p | FAM168A |
| hsa-miR-185-5p | PTCHD1 |
| hsa-miR-185-5p | TCF12 |
| hsa-miR-185-5p | MSI1 |
| hsa-miR-185-5p | CDC42 |
| hsa-miR-185-5p | ZCCHC3 |
| hsa-miR-185-5p | CAMK1G |
| hsa-miR-185-5p | SGMS1 |
| hsa-miR-185-5p | SF1 |
| hsa-miR-185-5p | TRIM67 |
| hsa-miR-185-5p | KLF7 |
| hsa-miR-185-5p | NFIX |
| hsa-miR-185-5p | CA10 |
| hsa-miR-185-5p | ANKRD34A |
| hsa-miR-185-5p | CBX5 |
| hsa-miR-185-5p | URM1 |
| hsa-miR-185-5p | BSN |
| hsa-miR-185-5p | CTSK |
| hsa-miR-185-5p | SPATA2 |
| hsa-miR-185-5p | LSAMP |
| hsa-miR-185-5p | IKZF4 |
| hsa-miR-185-5p | FAM76A |
| hsa-miR-185-5p | SMG7 |
| hsa-miR-185-5p | ABCC1 |
| hsa-miR-185-5p | IGF2R |
| hsa-miR-185-5p | RIMS4 |
| hsa-miR-185-5p | BEND3 |
| hsa-miR-185-5p | CAPZB |
| hsa-miR-185-5p | ZNF704 |
| hsa-miR-185-5p | PAK6 |
| hsa-miR-185-5p | CAMK2D |
| hsa-miR-185-5p | SIX3 |
| hsa-miR-185-5p | UHMK1 |
| hsa-miR-185-5p | SLC24A2 |
| hsa-miR-185-5p | GPR26 |
| hsa-miR-185-5p | EIF4B |
| hsa-miR-185-5p | PHLDB1 |
| hsa-miR-185-5p | DLG2 |
| hsa-miR-185-5p | ANKFY1 |
| hsa-miR-185-5p | FAIM2 |
| hsa-miR-185-5p | TRIM44 |
| hsa-miR-185-5p | MAT2A |
| hsa-miR-185-5p | SEC22A |
| hsa-miR-185-5p | USP3 |
| hsa-miR-501-3p | RNF38 |
| hsa-miR-501-3p | ANP32E |
| hsa-miR-501-3p | OTUD4 |
| hsa-miR-501-3p | ARIH1 |
| hsa-miR-501-3p | RPRD1B |
| hsa-miR-501-3p | SEC63 |
| hsa-miR-501-3p | KDM4A |
| hsa-miR-501-3p | KIAA0408 |
| hsa-miR-501-3p | PPP4R2 |
| hsa-miR-501-3p | ATXN1 |
| hsa-miR-501-3p | ADCY2 |
| hsa-miR-501-3p | JDP2 |
| hsa-miR-501-3p | TFDP2 |
| hsa-miR-501-3p | EXOC5 |
| hsa-miR-501-3p | SAMD12 |
| hsa-miR-501-3p | CSDE1 |
| hsa-miR-501-3p | DCLK1 |
| hsa-miR-501-3p | ESRRG |
| hsa-miR-501-3p | AFF4 |
| hsa-miR-501-3p | UCHL5 |
| hsa-miR-501-3p | PPP2R5E |
| hsa-miR-584-5p | AK7 |
| hsa-miR-584-5p | ROCK2 |
| hsa-miR-584-5p | ARL13B |
| hsa-miR-584-5p | GNG12 |
| hsa-miR-584-5p | PI15 |
| hsa-miR-584-5p | NEGR1 |
| hsa-miR-584-5p | CEP104 |
| hsa-miR-584-5p | GPBP1 |
| hsa-miR-584-5p | ZNF84 |
| hsa-miR-584-5p | ZNF585A |
| hsa-miR-584-5p | HOOK1 |
| hsa-miR-584-5p | TTC14 |
| hsa-miR-584-5p | MCPH1 |
| hsa-miR-584-5p | ZNF445 |
| hsa-miR-584-5p | HECW1 |
| hsa-miR-584-5p | TRAF3IP2 |
| hsa-miR-584-5p | ABL2 |
| hsa-miR-584-5p | ENAH |
| hsa-miR-584-5p | THBD |
| hsa-miR-584-5p | ODF2L |
| hsa-miR-584-5p | XPR1 |
| hsa-miR-584-5p | ZNF431 |
| hsa-miR-584-5p | MTRF1 |
| hsa-miR-584-5p | KPNB1 |
| hsa-miR-584-5p | PELI2 |
| hsa-miR-584-5p | ZNF529 |
| hsa-miR-584-5p | RBPJ |
| hsa-miR-584-5p | BTN3A1 |
| hsa-miR-584-5p | TMEM212 |
| hsa-miR-584-5p | PDE3A |
| hsa-miR-584-5p | POGLUT1 |
| hsa-miR-584-5p | GALNT15 |
| hsa-miR-584-5p | ZNF268 |
| hsa-miR-584-5p | ENTPD1 |
| hsa-miR-584-5p | FAM78B |
| hsa-miR-584-5p | FAM104B |
| hsa-miR-584-5p | SNTG1 |
| hsa-miR-584-5p | TTYH3 |
| hsa-miR-584-5p | KRAS |
| hsa-miR-584-5p | GATAD1 |
| hsa-miR-584-5p | SCAI |
| hsa-miR-584-5p | PHF6 |
| hsa-miR-584-5p | MMAA |
| hsa-miR-584-5p | CTXN2 |
| hsa-miR-584-5p | AVPR1A |
| hsa-miR-584-5p | DSG3 |
| hsa-miR-584-5p | GABRA4 |
| hsa-miR-584-5p | UBTD2 |
| hsa-miR-584-5p | EIF2AK1 |
| hsa-miR-584-5p | HBS1L |
| hsa-miR-584-5p | PEX5L |
| hsa-miR-1246 | SIAH3 |
| hsa-miR-1246 | DUSP18 |
| hsa-miR-1246 | SCN3A |
| hsa-miR-1246 | AMMECR1 |
| hsa-miR-1246 | MSR1 |
| hsa-miR-1246 | PSMA5 |
| hsa-miR-1246 | SPA17 |
| hsa-miR-1246 | PSD3 |
| hsa-miR-1246 | PLEKHG2 |
| hsa-miR-1246 | ANTXR2 |
| hsa-miR-1246 | FAM53C |
| hsa-miR-1246 | PMP2 |
| hsa-miR-1246 | TET2 |
| hsa-miR-1246 | CADM1 |
| hsa-miR-1246 | CBX5 |
| hsa-miR-1246 | GMNC |
| hsa-miR-1246 | ATRN |
| hsa-miR-3143 | PTPN4 |
| hsa-miR-3143 | CCDC117 |
| hsa-miR-3143 | SNX18 |
| hsa-miR-3143 | FZD5 |
| hsa-miR-3143 | BHLHE40 |
| hsa-miR-3143 | SLC16A7 |
| hsa-miR-3143 | NCEH1 |
| hsa-miR-3143 | ARMC8 |
| hsa-miR-3143 | SAMD8 |
| hsa-miR-3143 | KLF7 |
| hsa-miR-3143 | FUT9 |
| hsa-miR-3143 | HCN1 |
| hsa-miR-3143 | SLCO2A1 |
| hsa-miR-3143 | PANK1 |
| hsa-miR-3143 | BARD1 |
| hsa-miR-3143 | KCNN3 |
| hsa-miR-3143 | IRS2 |
| hsa-miR-3143 | LIMS1 |
| hsa-miR-3143 | RAB11FIP1 |
| hsa-miR-3143 | HSPA12A |
| hsa-miR-3143 | TWF1 |
| hsa-miR-3143 | CSDE1 |
| hsa-miR-3143 | SIPA1L2 |
| hsa-miR-3143 | HSPA4L |
| hsa-miR-3143 | ZBTB43 |
| hsa-miR-3143 | FAM169A |
| hsa-miR-3143 | ZMAT3 |
| hsa-miR-3143 | ATF7IP |
| hsa-miR-3143 | LRCH2 |
| hsa-miR-3143 | ZNF302 |
| hsa-miR-3143 | STEAP2 |
| hsa-miR-3143 | BICC1 |
| hsa-miR-3143 | PAIP2B |
| hsa-miR-3143 | CYP26B1 |
| hsa-miR-3143 | ANO6 |
| hsa-miR-4435 | PAX7 |
| hsa-miR-4435 | CORO2A |
| hsa-miR-4435 | ETNK2 |
| hsa-miR-4435 | C22orf23 |
| hsa-miR-4435 | STMN1 |
| hsa-miR-4435 | DAAM2 |
| hsa-miR-4435 | STK24 |
| hsa-miR-4435 | CLNK |
| hsa-miR-4435 | FOXN3 |
| hsa-miR-4435 | SKI |
| hsa-miR-4435 | UBE2H |
| hsa-miR-4435 | UVSSA |
| hsa-miR-4435 | ILDR2 |
| hsa-miR-4435 | FAM168A |
| hsa-miR-4435 | CLEC5A |
| hsa-miR-4435 | ELMOD3 |
| hsa-miR-4435 | MAPKBP1 |
| hsa-miR-4435 | XKR4 |
| hsa-miR-4435 | NSUN4 |
| hsa-miR-4435 | TFDP2 |
| hsa-miR-4435 | MMRN2 |
| hsa-miR-4435 | WDR5B |
| hsa-miR-4435 | DCUN1D3 |
| hsa-miR-4435 | STC1 |
| hsa-miR-4435 | ANK2 |
| hsa-miR-4435 | TPH1 |
| hsa-miR-4435 | ARFIP2 |
| hsa-miR-4435 | CLDN19 |
| hsa-miR-4435 | FOXP4 |
| hsa-miR-4435 | HCCS |
| hsa-miR-4435 | WNT2B |
| hsa-miR-4435 | DBN1 |
| hsa-miR-4435 | RANBP10 |
| hsa-miR-4435 | ACVR1B |
| hsa-miR-4435 | TOMM40L |
| hsa-miR-4435 | KCNB1 |
| hsa-miR-4435 | PPIL6 |
| hsa-miR-4435 | NPC2 |
| hsa-miR-4435 | ABI2 |
| hsa-miR-4435 | CBFA2T2 |
| hsa-miR-4435 | DRAM2 |
| hsa-miR-4435 | FAM122A |
| hsa-miR-4435 | SLC17A4 |
| hsa-miR-4435 | BMP2 |
| hsa-miR-4435 | PLEKHO2 |
| hsa-miR-4435 | KCNN3 |
| hsa-miR-4435 | BBX |
| hsa-miR-4435 | VASH2 |
| hsa-miR-4435 | FAM47E-STBD1 |
| hsa-miR-4435 | CALN1 |
| hsa-miR-4435 | DCAF10 |
| hsa-miR-4435 | MED19 |
| hsa-miR-4435 | CYP8B1 |
| hsa-miR-4435 | NRK |
| hsa-miR-4435 | TCN2 |
| hsa-miR-4435 | EPHB2 |
| hsa-miR-4435 | LRTM2 |
| hsa-miR-4435 | SOS1 |
| hsa-miR-4435 | SLC23A2 |
| hsa-miR-4435 | CASP10 |
| hsa-miR-4435 | PIK3R3 |
| hsa-miR-4435 | CDK6 |
| hsa-miR-4435 | DNAJC18 |
| hsa-miR-4435 | RBPMS2 |
| hsa-miR-4435 | DCAF16 |
| hsa-miR-4435 | EMX1 |
| hsa-miR-4435 | BACE1 |
| hsa-miR-4435 | DGKG |
| hsa-miR-4435 | CAPRIN1 |
| hsa-miR-4435 | PEA15 |
| hsa-miR-4435 | RAB3B |
| hsa-miR-4435 | RPAIN |
| hsa-miR-4435 | ETS1 |
| hsa-miR-4435 | C18orf54 |
| hsa-miR-4732-3p | ADCYAP1R1 |
| hsa-miR-4732-3p | ADAR |
| hsa-miR-4732-3p | LURAP1L |
| hsa-miR-4732-3p | SERINC3 |
| hsa-miR-4732-3p | BCL2L2 |
| hsa-miR-4732-3p | EFCAB14 |
| hsa-miR-4732-3p | PRICKLE2 |
| hsa-miR-4732-3p | LANCL3 |
| hsa-miR-4732-3p | ACSM2A |
| hsa-miR-4732-3p | PATZ1 |
| hsa-miR-4732-3p | ARID3B |
| hsa-miR-4732-3p | HIPK2 |
| hsa-miR-4732-3p | KPNB1 |
| hsa-miR-4732-3p | OAZ2 |
| hsa-miR-4732-3p | LIF |
| hsa-miR-4732-3p | RHOBTB2 |
| hsa-miR-4732-3p | FIGN |
| hsa-miR-4732-3p | ZFP62 |
| hsa-miR-4732-3p | HMGN2 |
| hsa-miR-4732-3p | EXTL3 |
| hsa-miR-4732-3p | NKPD1 |
| hsa-miR-4732-3p | ATXN1 |
| hsa-miR-4732-3p | DUSP26 |
| hsa-miR-4732-3p | RBM20 |
| hsa-miR-4732-3p | B4GALT6 |
| hsa-miR-4732-3p | TYSND1 |
| hsa-miR-4732-3p | CLOCK |
| hsa-miR-4732-3p | STXBP5L |
| hsa-miR-4732-3p | SUV39H1 |
| hsa-miR-4732-3p | SPATA5 |
| hsa-miR-4732-3p | KSR2 |
| hsa-miR-4732-3p | GGA2 |
| hsa-miR-4732-3p | SRF |
| hsa-miR-4732-5p | CCNY |
| hsa-miR-4732-5p | AHCYL2 |
| hsa-miR-4732-5p | UBE2W |
| hsa-miR-4732-5p | GSK3B |
| hsa-miR-4732-5p | PSMA5 |
| hsa-miR-4732-5p | FAM13A |
| hsa-miR-4732-5p | FZD5 |
| hsa-miR-4732-5p | SOCS5 |
| hsa-miR-4732-5p | EFR3A |
| hsa-miR-4732-5p | CCDC126 |
| hsa-miR-4732-5p | LPP |
| hsa-miR-4732-5p | MOB3C |
| hsa-miR-4732-5p | MRAP2 |
| hsa-miR-4732-5p | GRIN2C |
| hsa-miR-4732-5p | DBT |
| hsa-miR-4732-5p | SOX17 |
| hsa-miR-4732-5p | GLYCTK |
| hsa-miR-4732-5p | OXGR1 |
| hsa-miR-4732-5p | C21orf62 |
| hsa-miR-4732-5p | CDKN2AIP |
| hsa-miR-4732-5p | CDC42SE2 |
| hsa-miR-4732-5p | CARF |
| hsa-miR-4732-5p | DDX3X |
| hsa-miR-4732-5p | FOSL1 |
| hsa-miR-4732-5p | PARM1 |
| hsa-miR-4732-5p | POM121L12 |
| hsa-miR-4732-5p | AMMECR1 |
| hsa-miR-4732-5p | LSAMP |
| hsa-miR-4732-5p | YWHAG |
| hsa-miR-4732-5p | GDNF |
| hsa-miR-4732-5p | CPSF6 |
| hsa-miR-4732-5p | GABRB3 |
| hsa-miR-4732-5p | STS |
| hsa-miR-4732-5p | NHLH1 |
| hsa-miR-4732-5p | GJA5 |
| hsa-miR-4732-5p | AKAP7 |
| hsa-miR-4732-5p | TMEM237 |
| hsa-miR-4732-5p | APOA5 |
| hsa-miR-4732-5p | RRP8 |
| hsa-miR-4732-5p | SLFN13 |
| hsa-miR-4732-5p | RAVER2 |
| hsa-miR-4732-5p | CTBP2 |
| hsa-miR-4732-5p | GIT2 |
| hsa-miR-34a-5p | KCNK3 |
| hsa-miR-34a-5p | PTPN4 |
| hsa-miR-34a-5p | KCNQ3 |
| hsa-miR-34a-5p | MTMR9 |
| hsa-miR-34a-5p | GABRA3 |
| hsa-miR-34a-5p | RAB43 |
| hsa-miR-34a-5p | FAM126B |
| hsa-miR-34a-5p | ZCCHC17 |
| hsa-miR-34a-5p | EVI5L |
| hsa-miR-34a-5p | UHRF2 |
| hsa-miR-34a-5p | C8orf37 |
| hsa-miR-34a-5p | FOXJ2 |
| hsa-miR-34a-5p | ISY1-RAB43 |
| hsa-miR-34a-5p | RORA |
| hsa-miR-34a-5p | SIDT2 |
| hsa-miR-34a-5p | MYRIP |
| hsa-miR-34a-5p | ELL2 |
| hsa-miR-129-2-3p | MMP16 |
| hsa-miR-129-2-3p | ANKRD52 |
| hsa-miR-129-2-3p | USP13 |
| hsa-miR-129-2-3p | PTPN1 |
| hsa-miR-138-5p | USP47 |
| hsa-miR-138-5p | RHOC |
| hsa-miR-138-5p | DENND1A |
| hsa-miR-138-5p | AHCYL2 |
| hsa-miR-138-5p | NEBL |
| hsa-miR-138-5p | TCF4 |
| hsa-miR-138-5p | RDH8 |
| hsa-miR-138-5p | PSMF1 |
| hsa-miR-138-5p | ZNF275 |
| hsa-miR-138-5p | PPARGC1A |
| hsa-miR-138-5p | KIAA0930 |
| hsa-miR-138-5p | ARFGEF2 |
| hsa-miR-138-5p | PWWP2A |
| hsa-miR-138-5p | SOGA1 |
| hsa-miR-138-5p | FEM1C |
| hsa-miR-138-5p | NKAIN1 |
| hsa-miR-138-5p | SLC22A23 |
| hsa-miR-138-5p | EID1 |
| hsa-miR-138-5p | VPS37A |
| hsa-miR-138-5p | CALN1 |
| hsa-miR-138-5p | NSFL1C |
| hsa-miR-138-5p | ILDR2 |
| hsa-miR-138-5p | UNC5D |
| hsa-miR-138-5p | KANK1 |
| hsa-miR-138-5p | ERI1 |
| hsa-miR-138-5p | VPS26A |
| hsa-miR-138-5p | LYPLA1 |
| hsa-miR-138-5p | CTDSPL2 |
| hsa-miR-138-5p | THAP11 |
| hsa-miR-138-5p | ZNF148 |
| hsa-miR-138-5p | MFAP3 |
| hsa-miR-138-5p | FOXC1 |
| hsa-miR-138-5p | LSM14A |
| hsa-miR-138-5p | PAPPA |
| hsa-miR-138-5p | EFNB3 |
| hsa-miR-138-5p | STOX2 |
| hsa-miR-138-5p | FAM169A |
| hsa-miR-138-5p | RIMS3 |
| hsa-miR-138-5p | SZRD1 |
| hsa-miR-138-5p | PDIK1L |
| hsa-miR-138-5p | XPR1 |
| hsa-miR-138-5p | CREB3L2 |
| hsa-miR-138-5p | VEZF1 |
| hsa-miR-138-5p | RARA |
| hsa-miR-138-5p | NFIB |
| hsa-miR-138-5p | GALNTL6 |
| hsa-miR-138-5p | NPPC |
| hsa-miR-138-5p | HIF1AN |
| hsa-miR-138-5p | GAS7 |
| hsa-miR-138-5p | DESI2 |
| hsa-miR-138-5p | TSR1 |
| hsa-miR-138-5p | ZBTB44 |
| hsa-miR-138-5p | LHFPL3 |
| hsa-miR-138-5p | TRPS1 |
| hsa-miR-138-5p | PHF21A |
| hsa-miR-152-5p | FAXC |
| hsa-miR-152-5p | ISY1 |
| hsa-miR-152-5p | GDAP2 |
| hsa-miR-152-5p | HOXD13 |
| hsa-miR-152-5p | PHKA1 |
| hsa-miR-152-5p | SLCO1A2 |
| hsa-miR-200a-3p | FAM160B1 |
| hsa-miR-200a-3p | AKAP11 |
| hsa-miR-200a-3p | FBXW2 |
| hsa-miR-200a-3p | GPR137C |
| hsa-miR-200a-3p | PPM1L |
| hsa-miR-200a-3p | STX16 |
| hsa-miR-200a-3p | GAB1 |
| hsa-miR-342-5p | CREB3L1 |
| hsa-miR-342-5p | APBB2 |
| hsa-miR-342-5p | SUMO1 |
| hsa-miR-342-5p | FBXO46 |
| hsa-miR-342-5p | LIMD2 |
| hsa-miR-342-5p | ZFHX2 |
| hsa-miR-342-5p | IL2RG |
| hsa-miR-342-5p | ZFP3 |
| hsa-miR-342-5p | MXRA8 |
| hsa-miR-342-5p | TUSC2 |
| hsa-miR-342-5p | ARHGEF18 |
| hsa-miR-342-5p | GREM1 |
| hsa-miR-342-5p | PTPRN2 |
| hsa-miR-342-5p | PCBP4 |
| hsa-miR-342-5p | SH2D3C |
| hsa-miR-342-5p | MKNK2 |
| hsa-miR-342-5p | EFNA5 |
| hsa-miR-342-5p | MEX3A |
| hsa-miR-342-5p | IQSEC3 |
| hsa-miR-342-5p | TIMM17B |
| hsa-miR-342-5p | TEX19 |
| hsa-miR-342-5p | XIRP1 |
| hsa-miR-342-5p | IRF6 |
| hsa-miR-342-5p | TPBGL |
| hsa-miR-342-5p | FURIN |
| hsa-miR-342-5p | LMOD1 |
| hsa-miR-342-5p | SZRD1 |
| hsa-miR-342-5p | AFF2 |
| hsa-miR-342-5p | IGF2 |
| hsa-miR-342-5p | BCL2L1 |
| hsa-miR-342-5p | ARF3 |
| hsa-miR-342-5p | PFN1 |
| hsa-miR-501-5p | VGLL3 |
| hsa-miR-501-5p | METTL21A |
| hsa-miR-501-5p | UBN2 |
| hsa-miR-501-5p | ZBTB20 |
| hsa-miR-501-5p | IL18BP |
| hsa-miR-501-5p | PSMD5 |
| hsa-miR-501-5p | ZNF568 |
| hsa-miR-501-5p | LIN28B |
| hsa-miR-501-5p | PI15 |
| hsa-miR-501-5p | FGF12 |
| hsa-miR-501-5p | NR4A3 |
| hsa-miR-501-5p | PANK3 |
| hsa-miR-501-5p | USP38 |
| hsa-miR-501-5p | BRI3BP |
| hsa-miR-501-5p | OGFRL1 |
| hsa-miR-501-5p | MBTD1 |
| hsa-miR-501-5p | PPP1CB |
| hsa-miR-501-5p | GAS2 |
| hsa-miR-501-5p | PPP6R3 |
| hsa-miR-501-5p | PLP1 |
| hsa-miR-501-5p | MEF2C |
| hsa-miR-501-5p | AMER2 |
| hsa-miR-501-5p | LPP |
| hsa-miR-501-5p | NUFIP2 |
| hsa-miR-501-5p | MBNL2 |
| hsa-miR-501-5p | ZBTB38 |
| hsa-miR-501-5p | CROT |
| hsa-miR-501-5p | SSR3 |
| hsa-miR-501-5p | SRR |
| hsa-miR-501-5p | GABRB2 |
| hsa-miR-501-5p | DDX52 |
| hsa-miR-501-5p | PAK3 |
| hsa-miR-501-5p | KLF12 |
| hsa-miR-501-5p | RIMKLB |
| hsa-miR-501-5p | DENND5B |
| hsa-miR-501-5p | SRGN |
| hsa-miR-501-5p | MTMR9 |
| hsa-miR-501-5p | RNF165 |
| hsa-miR-501-5p | PRPF40A |
| hsa-miR-501-5p | PRDX3 |
| hsa-miR-501-5p | MSI2 |
| hsa-miR-501-5p | DCP2 |
| hsa-miR-501-5p | KRR1 |
| hsa-miR-501-5p | CTDSP1 |
| hsa-miR-501-5p | EDEM1 |
| hsa-miR-501-5p | ARHGEF33 |
| hsa-miR-501-5p | UBA5 |
| hsa-miR-501-5p | SLC35A3 |
| hsa-miR-501-5p | RBM8A |
| hsa-miR-501-5p | MAP2K1 |
| hsa-miR-501-5p | LPAR1 |
| hsa-miR-501-5p | PNN |
| hsa-miR-501-5p | PARM1 |
| hsa-miR-501-5p | PI4KB |
| hsa-miR-501-5p | EIF3M |
| hsa-miR-501-5p | ZBTB18 |
| hsa-miR-501-5p | DRAM2 |
| hsa-miR-501-5p | SYT7 |
| hsa-miR-501-5p | ZC3H12C |
| hsa-miR-501-5p | NR2F2 |
| hsa-miR-501-5p | ADARB1 |
| hsa-miR-501-5p | VKORC1L1 |
| hsa-miR-501-5p | DCLRE1A |
| hsa-miR-501-5p | TRNT1 |
| hsa-miR-501-5p | MRPS16 |
| hsa-miR-501-5p | SSR1 |
| hsa-miR-501-5p | ZC3H4 |
| hsa-miR-501-5p | STXBP1 |
| hsa-miR-501-5p | HTR2A |
| hsa-miR-501-5p | EPM2AIP1 |
| hsa-miR-501-5p | MPP7 |
| hsa-miR-501-5p | LPGAT1 |
| hsa-miR-501-5p | CACHD1 |
| hsa-miR-501-5p | HSPA4L |
| hsa-miR-501-5p | NR3C1 |
| hsa-miR-501-5p | NPR3 |
| hsa-miR-501-5p | MED6 |
| hsa-miR-501-5p | VPS13A |
| hsa-miR-501-5p | OSBPL6 |
| hsa-miR-501-5p | DCDC2 |
| hsa-miR-501-5p | ZNF701 |
| hsa-miR-501-5p | IKZF2 |
| hsa-miR-501-5p | NFASC |
| hsa-miR-501-5p | PHF6 |
| hsa-miR-501-5p | WIPF2 |
| hsa-miR-501-5p | CHST9 |
| hsa-miR-501-5p | TRIM39 |
| hsa-miR-501-5p | PDE11A |
| hsa-miR-501-5p | KCNRG |
| hsa-miR-501-5p | SRSF2 |
| hsa-miR-501-5p | CEP41 |
| hsa-miR-501-5p | MLANA |
| hsa-miR-501-5p | RBM27 |
| hsa-miR-501-5p | GATAD2B |
| hsa-miR-4777-3p | PDE4D |
| hsa-miR-4777-3p | CDC23 |
| hsa-miR-4777-3p | ZNF830 |
| hsa-miR-4777-3p | DGKH |
| hsa-miR-4777-3p | MARK1 |
| hsa-miR-4777-3p | CREB5 |
| hsa-miR-4777-3p | SMC2 |
| hsa-miR-4777-3p | WNK3 |
| hsa-miR-4777-3p | GAS7 |
| hsa-miR-6503-5p | AGO2 |
| hsa-miR-6503-5p | TLK1 |
| hsa-miR-6503-5p | DDI2 |
| hsa-miR-6503-5p | RUNX1T1 |
| hsa-miR-6503-5p | GORASP2 |
| hsa-miR-6503-5p | TMEM26 |
| hsa-miR-6503-5p | ISY1 |
| hsa-miR-6503-5p | C3orf18 |
| hsa-miR-6503-5p | NCALD |
| hsa-miR-6503-5p | KSR2 |
| hsa-miR-6503-5p | CBX5 |
| hsa-miR-6503-5p | FOXO3 |

Abbreviations: DEMs, differentially expressed microRNAs
